# The use of advanced medical technologies at home: a systematic review of the literature

**DOI:** 10.1186/s12889-018-5123-4

**Published:** 2018-02-26

**Authors:** Ingrid ten Haken, Somaya Ben Allouch, Wim H. van Harten

**Affiliations:** 1 Saxion University of Applied Sciences, Research Group Technology, Health & Care (TH&C), P.O. Box 70.000, 7500 KB Enschede, The Netherlands; 2 Department Health Technology & Services Research (HTSR), University of Twente, Faculty Behavioural, Management and Social Sciences (BMS), Ravelijn 5246, P.O. Box 217, 7500 AE Enschede, The Netherlands; 3 Rijnstate General Hospital, Arnhem, The Netherlands

**Keywords:** Home health nursing, Medical technologies, Patient safety, Quality of health care, Systematic review, Trends

## Abstract

**Background:**

The number of medical technologies used in home settings has increased substantially over the last 10–15 years. In order to manage their use and to guarantee quality and safety, data on usage trends and practical experiences are important. This paper presents a literature review on types, trends and experiences with the use of advanced medical technologies at home.

**Methods:**

The study focused on advanced medical technologies that are part of the technical nursing process and ‘hands on’ processes by nurses, excluding information technology such as domotica. The systematic review of literature was performed by searching the databases MEDLINE, Scopus and Cinahl. We included papers from 2000 to 2015 and selected articles containing empirical material.

**Results:**

The review identified 87 relevant articles, 62% was published in the period 2011–2015. Of the included studies, 45% considered devices for respiratory support, 39% devices for dialysis and 29% devices for oxygen therapy. Most research has been conducted on the topic ‘user experiences’ (36%), mainly regarding patients or informal caregivers. Results show that nurses have a key role in supporting patients and family caregivers in the process of homecare with advanced medical technologies and in providing information for, and as a member of multi-disciplinary teams. However, relatively low numbers of articles were found studying nurses perspective.

**Conclusions:**

Research on medical technologies used at home has increased considerably until 2015. Much is already known on topics, such as user experiences; safety, risks, incidents and complications; and design and technological development. We also identified a lack of research exploring the views of nurses with regard to medical technologies for homecare, such as user experiences of nurses with different technologies, training, instruction and education of nurses and human factors by nurses in risk management and patient safety.

## Background

As a result of demographic changes and the rapidly increasing number of older patients, there is a need for cost savings and health reforms, which include an increased move from inpatient to outpatient care in most industrialized countries over the last 10–15 years [1, 2]. As a consequence, the transfer of advanced medical devices into home settings was considerable and it is expected that there will be a further increase in the near future [1–7].

When ‘an increase’ in the number of medical technologies used at home is mentioned, it is not clear which and how many technologies are involved. Today, there are an estimated 500,000 different kinds and types of medical devices available on the world market [8, 9]. The European Commission (EC) publishes data regarding legislation and regulations for medical devices, but the actual figures for medical technologies in outpatient practice are not available [10]. The U.S. National Center for Health Statistics (NCHS) stated that technologies have shifted from hospitals into the home, but it too does not illustrate its findings with statistics [11]. We searched for data with regard to the actual number of medical technologies used in home settings and it proved difficult to find any systematic data sets available throughout the international landscape.

An important condition for the application of medical technology in the home setting is that quality of care and patient safety must be guaranteed [6]. From a historical perspective medical technologies were designed for hospital settings [12, 13]. This means that specific factors regarding the implementation and use at home now need to be taken into account [7, 14, 15]. In general, risks with medical technologies can be classified regarding (a) environmental factors; (b) human factors and (c) technological factors [16]. Human factors, however, are very important in patient safety in both hospital and in home settings [1, 6, 12]. For example, a major risk factor is the number of users and handovers in the chain of care. In home settings, a sometimes impressive number of different users of medical technology, often with various levels of training, instruction or education, are involved. Although patient empowerment moves control to the patient and/or relatives, an important user group is that of professional nurses. Understanding user experiences and information about adverse events and near incidents are important aspects for developing knowledge regarding implementation and use in home care setting. Sharing this knowledge can support patients and caregivers, and especially nurses in their professional work and will also contribute to patient safety and quality of care.

Therefore, there is a need to address the question first, which types of technologies are used at home; second, how frequently are they used and third, what trends can be distinguished. Additional research questions are whether there are any scientific data regarding particular user experiences; training, instruction and education; safety and risks, and finally, what can be concluded about the role of nurses in using medical technologies in the home environment. The objective of this paper therefore is to present a systematic literature search on the international state of art concerning various aspects of the use of advanced medical technologies at home.

### Definitions

First, we want to clarify some definitions. In general, ‘health technology’ refers to the application of organized knowledge and skills in the form of devices, medicines, vaccines, procedures and systems developed to solve a health problem and improve quality of life [17]. The World Health Organization [8] uses the definition of ‘medical device’ as ‘An article, instrument, apparatus or machine that is used in the prevention, diagnosis or treatment of illness or disease, or for detecting, measuring, restoring, correcting or modifying the structure or function of the body for some health purpose …….’. A specification for a home use medical device is: ‘A medical device intended for users in any environment outside of a professional healthcare facility. This includes devices intended for use in both professional healthcare facilities and homes’ [18].

The landscape of medical devices is diverse with technologies varying from relatively simple to very complex devices. Wagner et al. [19] stated that ‘high-tech dependency’ (for children) matches with ‘technology-dependency’ if it concerns ‘a medical device to compensate for the loss of a vital bodily function and substantial and ongoing nursing care to avert death or further disability’. ‘The needs of these patients may vary from the continuous assistance of a device and highly trained caretaker to less frequent treatment and intermittent nursing care’ [20]. Although patients dependent of advanced medical technologies at home are often medically stable, they sometimes have high technical needs and may be expected to need long-term recovery. They also require skilled nursing [21] and a considerable degree of advanced decision making, planning, training and oversight [22]. An overall definition of ‘advanced medical technology’ is: ‘Medical devices and software systems that are complex, provide critical patient data, or that directly implement pharmacologic or life-support processes whereby inadvertent misuse or use error could present a known probability of patient harm’ [23]. Examples of advanced medical technologies used at home include ventilators for respiratory support, systems for haemo- or peritoneal dialysis and infusion pumps to provide nutrition or medication.

In the Netherlands, the National Institute for Public Health and the Environment (RIVM) [24] uses the following definition:

*Advanced medical technology or high-tech technology in the home setting is defined as technology that is part of the technical skills in nursing and meets the following conditions:*

- *technology that is advanced or high-tech, for example equipment with a plug, an on/off switch, an alarm button and a pause button;*
- *technology that had been applied formerly only in hospital care, but that is now also often applied in home settings;*
- *technology that can be categorized as ‘supporting physiological functions’, ‘administration’ or ‘monitoring’.*

Within the Dutch classification of advanced medical technologies 19 different devices are identified (see Table 1), which will be used in this review as a basis to categorize the technologies. It is a classification format in which specific advanced technologies are defined. Terms as ‘advanced medical technology’ (from now on abbreviated as AMT) will be used consistently as synonyms for ‘complex medical technology’ and ‘high-tech medical technology’. The term ‘technology’ will be used in the meaning of ‘device’ or ‘equipment’. The target is on technologies that are instrumental and ‘hands on’ use by nurses in the care for patients. This means that information technology (IT) based technologies as domotica (automation for a home) are not part of the study.

**Table 1:** Classification of advanced medical technologies in the Netherlands according to the National Institute for Public Health and the Environment (RIVM) [24]

Technologies with regard to:	
Supporting physiological functions:	
1. Respiratory support	
2. Sleep apnea treatment	
3. Suction devices	
4. Oxygen therapy	
5. Dialysis	
6. Vacuum assisted wound closure	
7. Decubitus treatment	
8. External electrostimulation	
9. Continuous passive motion	
10. Skeletal traction	
11. Patient lifting hoists	
Administration:	
12. Infusion therapy	
13. Insulin pump therapy	
14. Parenteral nutrition	
15. Enteral nutrition	
16. UV therapy	
17. Nebulizer	
Monitoring:	
18. Fetal cardiotocography	
19. Respiratory and circulatory monitoring	

## Methods

### Eligibility and search strategy

The systematic review of the literature was conducted early 2016. Key concepts for the review were ‘medical technologies’ or ‘medical devices’, and ‘home settings’. The concept of ‘home settings’ is related to the terms ‘home nursing’ and ‘home care service’, of which the stem is ‘home’. Combining the key concepts provided the search string: (‘medical technology’ OR ‘medical device’). As domotica is not part of the study, the search string was extended with: AND NOT (eHealth OR telecare OR telemedicine). The exact search string is (“medical technology” OR “medical devices”) AND home AND NOT (ehealth OR telecare OR telemedicine). Online databases MEDLINE, Scopus and Cinahl were searched electronically using the search string to obtain data.

### Inclusion and exclusion criteria

Criteria for selection were defined prior to the search process. General criteria for inclusion were:

- Year of publication: 2000–2015.
- An abstract or an article (with or without abstract) has to be available, containing reference to AMT information.
- The article is published in English, German, French or Dutch/Flemish language.
- If medical technology is cited, it has to conform to the definition of ‘advanced medical technology’ [24].
- The abstract or the article has to contain empirical material. For the purpose of this review, ‘empirical material’ has been defined as: AMT which is designed for the home setting, or where the design or choices took into account the setting of the home, or where the medical technology has been tested for the home or if the medical technology is already on the market and being used in the home setting.

For further selection, inclusion criteria related to the key concepts for title and abstract were applied, such as ‘advanced medical technology’, ‘high-tech medical technology’, ‘home-centred health-enabling technology’ and ‘care at home’. The classification of the RIVM (see Table 1) has been taken as a basis to categorize technologies in this review. Domotica and telemonitoring technologies scored under ‘monitoring’, such as fetal cardiotocography, and respiratory and circulatory monitoring, were left out. If the abstract or article was about electronic health records, ‘smart home’, ambient intelligence, pervasive computing, software of devices, smartphone or surgical robots, the article was also removed from selection. Technologies as ‘VAD (ventricular assist device)’, ‘dental devices’ and ‘AED (automatic external defibrillator)’ were not seen as part of the technical nursing process and these records were left out as well. Studies conducted in the hospital, hospice or nursing home settings were also excluded. An overview of all inclusion and exclusion criteria can be found in Table 2.

**Table 2:** Inclusion and exclusion criteria for title and abstract and/or

	Inclusion	Exclusion
Title	Advanced medical technology (−ies) Medical technology (−ies) Medical device(s) High-tech medical technology (−ies) High-tech home care Home Homecare Home health care Home-based care Home-based technology (−ies) Home-centered health-enabling technology (−ies) Care at home Care in the home AND *Inclusion criteria for abstract below*	eHealth Telehealth Telenursing Telemedicine Telemedical system Telehomecare Telecare Teleconsultation Ambient assisted environment iPad technology VAD (ventricular assist device) Dental devices ECG (electrocardiogram) AED (automatic external defibrillator) Hospital Hospice Nursing home
Abstract and/or article	Year of publication: 2000–2015 1. Respiratory support (RIVM) Respirator Respiratory support Respiratory therapy Ventilator Ventilator-assisted Mechanical ventilation Support ventilation Invasive ventilation Non-invasive ventilation Non-invasive mechanical ventilation Continuous positive airway pressure (CPAP) Bilevel positive airway pressure (BPAP, BiPAP) Negative pressure ventilation (NVP) Mechanical in-exsufflation 2. Sleep apnea treatment (RIVM) Sleep apnea treatment device (Positive) airway pressure device (PAP) 3. Suction devices (RIVM) Suction machine Suction apparatus Airway suction device Digital suction Mucus removal 4. Oxygen therapy (RIVM) Oxygen therapy Long-term oxygen cylinder Long-term oxygen ventilator Supplemental oxygen Oxygen conserver Oxygen concentrator Oxygen tank 5. Dialysis (RIVM) Haemo dialysis Hemo dialysis Peritoneal dialysis Peritoneal catheter drainage system Peritoneal automatic delivery system CAPD (Continuous Ambulatory Peritoneal Dialysis) APD (Automated Peritoneal Dialysis) Dialysis machine Sorbent dialysis 6. Vacuum assisted wound closure (RIVM) Negative-pressure wound therapy system VAC- therapy NPWT Vacuum assisted wound closure 7. Decubitus treatment (RIVM) Pressure ulcer treatment Decubitus treatment Decubitus mattress Pressure relief mattress 8. External electrostimulation (RIVM) (External) electrostimulation Electrical stimulation TENS Nerve stimulation Transcutaneaous electrical stimulation to treat slow-transit constipation 9. Continuous passive motion (RIVM) Continuous passive motion Motion therapy 10.Skeletal traction (RIVM) (Skeletal) traction Tension 11.Patient lifting hoists (RIVM) Patient lift Patient hoist Transfer device 12. Infusion therapy (RIVM) Infusion pump Infusion therapy Central venous catheter Central venous line Port a cath PICC (perifally inserted central catheter) Intravenous medication Intravenous therapy Intravenous chemotherapy Analgesia pump PCA-pump (patient controlled analgesia pump) Indwelling venous catheter 13. Insulin pump therapy (RIVM) Insulin pump therapy Insulin infusion 14. Parenteral nutrition (RIVM) Parenteral nutrition Parenteral feeding Intravenous nutrition 15. Enteral nutrition (RIVM) Enteral nutrition Tube feeding / feeding tube Feeding pump Enteral feeding PEG-tube (percutaneous endoscopic gastrostomy)	- If no abstract and no article available - If the title is in English, but the article is written not in English, German, French or Dutch/ Flemish. - If about medical technologies, but not about the application in the setting of the home. - If the abstract or article contains no empirical material. For the purpose of this review, ‘empirical material’ is defined as: • medical technology which is designed for the home setting, or • where the design or choices took into account the setting of the home, or • where the medical technology has been tested for the home and • if the medical technology is already on the market or being used in the home setting. - If the abstract or article is about: • electronic health records • ‘smart home’ • ambient intelligence • pervasive computing • software of devices • smart phone • (surgical) robots - If not conform the definition of RIVM (2013, page 15) of ‘complex medical technology’. *Advanced medical technology or high-tech technology in the home setting is defined as technology that is part of the technical skills in nursing and meets the following conditions:* • *technology that is advanced or high-tech, i.e. equipment with a plug, a switch on/off button, alarm button, pause button etc.;* • *technology that had been applied formerly in hospital care (‘intramural’), but that is applied also often in home settings nowadays;* • *technology that can be categorized as ‘supporting physiological functions’, ‘administration’ or ‘monitoring’.*
	PEGJ-tube (percutaneous endoscopic gastrostomy, jejeunum) Percutaneous gastrostomy tube Jejeunostomy tube Gastrostomy feeding 16. UV therapy (RIVM) UV therapy Ultraviolet therapy Phototherapy 17. Nebulizer (RIVM) Nebulizer	18. Fetal cardiotocography (RIVM) Electronic fetal monitoring Fetal monitoring Cardiotocography 19. Respiratory and circulatory monitoring (RIVM) Capnography Respiratory monitoring Circulatory monitoring Pulse oximeter Electrocardiography

### Screening process

**Fig. 1:**
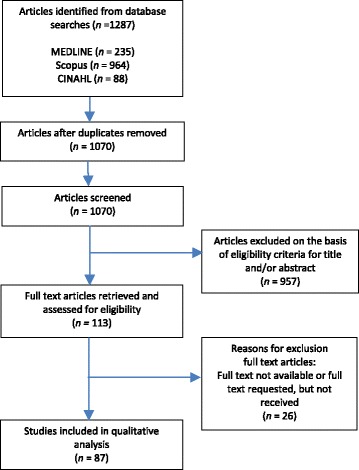
PRISMA flowchart

The search in the online databases using the search string, identified a total of 1287 references. After checking for duplicates, 1070 articles remained. Those articles were reviewed by a reviewer for titles and abstracts on basis of the inclusion and exclusion criteria. A double check was performed by two reviewers, who independently screened random samples of 20% of the articles. There was an initial agreement of 88%. In case of disagreement about the inclusion of an article, the decision was based on a joint discussion by all three reviewers to an agreement of 100% and the resulting screening policy was applied to the rest of the abstracts. Based on the selected titles and/or abstracts, articles were retrieved or requested in full text and assessed for eligibility. Some articles were excluded from further study, for reasons of ‘full text not available’ or the article contained no empirical material. Finally, 87 studies remained which were included in the analysis (see Table 3). A graphical representation of the screening process has been included in Fig. 1.

**Table 3:** Characteristics of included studies

Study	Country of study	Study design and sample	Medical technologies	Medical diagnosis	Content
Agar, J.W.M., Perkins, A., Tjipto, A., 2012. [96]	Australia	Separately metered and serially measured independent draws of each dialysis machine plus reverse osmosis pairing. *n* = 4 (home dialysis equipment combinations)	Hemodialysis	No medical diagnosis mentioned	Evaluation of solar-assisted hemodialysis.
Alsaleh, F.M., Smith, F.J., Thompson, R., Al-Saleh, M.A., Taylor, K.M., 2014. [32]	UK	Cross-sectional face-to-face semi-structured interviews; Qualitative and quantitative approaches for data analysis *n* = 34 (patients, children/young people) *n* = 38 (parents)	Insulin pump therapy	Type 1 diabetes mellitus	Examination of the impact of switching from multiple daily injections to insulin pumps on the glycaemic control and daily lives of children/young people and their families.
Amin, R.S., Fitton, C.M., 2003. [104]	USA		Long-term mechanical ventilation; Tracheostomy	Chronic respiratory failure (CRF) as indicated by hypoxemia and or hypercapnia; Chronic progressive lung diseases; Neuromuscular disorders; Congenital muscular dystrophy; Non-progressive chronic parenchymal lung disease; Congenital central hypoventilation syndrome (CCHS); Multiple genetic syndromes such as myelomeningocele with Arnold Chiari malformation, skeletal dysplasia, Möbius syndrome, Joubert and Prader-Willi syndromes, and inborn errors of metabolism such as pyruvate dehydrogenase complex deficiency, Leigh’s disease, and carnitine deficiency, could be associated with central hypoventilation; Bronchopulmonary dysplasia (BPD); Chest wall dysfunction such as asphyxiating thoracic dystrophy, short limb dwarfism, giant omphalocele; Idiopathic scoliosis	An overview of indications for use of home mechanical ventilation, different methods and modes of mechanical ventilation, ventilator settings and outcomes of long-term mechanical ventilation in children.
Ao, P., Sebastianski, M., Selvarajah, V., Gramlich, L., 2015. [83]	Canada	Retrospective chart review *n* = 560 (patients; *n* = 64 J-tube; *n* = 496 PEG tube)	Percutaneous endoscopic gastrostomy (PEG) tubes; Jejunostomy tubes (J-tubes)	Esophageal/gastric cancer; Head and neck cancer; Stroke; Neurologic; Other	Comparison of complication rates, types, and average tube patency between jejunostomy tubes and percutaneous gastrostomy tubes in a Regional Home Enteral Nutrition Support Program.
Bezruczko, N., Chen, S.P., Hill, C.D., Chesniak, J.M., 2009. [45]	USA	Functional Caregiving (FC); Survey methods guided by a caregiver content matrix validated by content and clinical reviews; Survey forms, questionnaires *n* = 53 (mothers)	Tracheostomy; Tracheostomy/ ventilator; BiPAP/CPAP	No medical diagnosis mentioned	Development of an objective, linear measure of mothers’ confidence to care for children assisted with medical technology in their homes.
Bezruczko, N., Chen, S.P., Hill, C.D., Chesniak, J.M., 2011. [46]	USA	Functional Caregiving (FC); Survey forms, questionnaires *n* = 53 (mothers)	Tracheostomy; Tracheostomy/ ventilator; BiPAP/CPAP	No medical diagnosis mentioned	Development of an objective, linear measure of mothers’ confidence to care for children assisted with medical technology in their homes.
Bortolussi, R., Zotti, P., Conte, M., Marson, R., Polesel, J., Colussi, A., Piazza, D., Tabaro, G., Spazzapan, S., 2015. [33]	Italy	Prospective observational study; Questionnaire; Structured interview; Monitoring form (filled in by a nurse) *n* = 48 (patients)	Peripherally inserted central venous catheters (PICCs); Midline catheters	Pancreatic cancer; Stomach cancer; Other miscellaneous cancer; Non-neoplastic diseases	Evaluation of distress and pain perceived by patients during the positioning of a PICC or midline catheter, both in the home and hospice settings, and the perceived quality of life.
Bostelman, R., Ryu, J.-C., Chang, T., Johnson, J., Agrawal, S.K., 2010. [93]	USA	Static stability tests; Dynamic stability tests; Method for autonomous maneuvers tested in simulation and experiments	Home Lift, Position and Rehabilitation (HLPR) Chair	No medical diagnosis mentioned	Evaluation of an advanced patient lift and transfer device for the home.
Brooks, D., King, A., Tonack, M., Simson, H., Gould, M., Goldstein, R., 2004. [29]	Canada	Study design based on grounded theory; Semi-structured interviews *n* = 26 (patients)	Long-term mechanical ventilation	Neuromuscular diseases: Polio; Amyotrophic lateral sclerosis (ALS); Cerebral palsy (CP); Duchenne muscular dystrophy (DMD); Muscular dystrophy (MD); Spinal cord injury (SCI); Spinal muscular atrophy (SMA); Transverse myelitis (TM)	Identify user perspectives on the issues that impact the quality of the daily lives of ventilator-assisted individuals living in the community.
Brown, K. A., Bertolizio, G., Leone, M., Dain, S.L., 2012. [100]	Canada	Review	Noninvasive ventilation (NIV)	Chronic stable respiratory failure; Obstructive sleep apnea	An overview of the indications for home NIV therapy, of the medical devices currently available to deliver it, and a specific discussion of the management conundrums confronting anesthesiologists.
Buchman, A.L., Opilla, M., Kwasny, M., Diamantidis, T.G., Okamoto, R., 2014. [63]	USA	Retrospective evaluation of patient records *n* = 143 (patients; *n* = 125 adults; *n* = 18 children)	Home parenteral nutrition (HPN)	Short bowel syndrome (SBS); Motility disorders; Other	Identification of risk factors for the development of catheter-related bloodstream infections (CRBSI) in patients receiving home parenteral nutrition.
Chatburn, R.L., 2009. [86]	USA	Review	Noninvasive ventilation (NIV)	Chronic obstructive pulmonary disease (COPD); Acute cardiogenic pulmonary edema; Hematologic malignancy; Bone marrow or solid-organ transplant; AIDS	Provision of the basis for a simple procedure for selecting the most appropriate NIV technology for the patient and the environment of care.
Craig, G.M., Scambler, G., Spitz, L., 2003. [44]	UK	Qualitative research study; Semi-structured in-depth interview *n* = 22 (parents of 22 children)	Gastrostomy feeding	Severe neuro-developmental disabilities: Cerebral palsy; Syndrome of chromosomal or genetic origin; Unconfirmed diagnoses	A study of parental perceptions of gastrostomy feeding before surgery to examine the factors parents consider when gastrostomy feeding is recommended and to identify the need for support.
Davenport, A., 2015. [64]	UK	Review	Hemodialysis	No medical diagnosis mentioned	Complications of hemodialysis treatments due to dialysate contamination and composition errors, and how to recognize them promptly to provide appropriate management and minimize patient harm.
dos Santos-Fontes, R.L., Ferreiro de Andrade, K.N., Sterr, A., Conforto, A.B., 2013. [62]	Brazil	Experimental design Pilot randomized double-blinded clinical trial, Perform tasks of the Jebsen-Taylor Test (JTT), Measurement in time, A written log by patients *n* = 20 (patients)	Repetitive peripheral nerve stimulation (RPSS)	Stroke	A proof-of-principle study: Home-based nerve stimulation to enhance effects of motor training in patients in the chronic phase after stroke.
Dubois, P., Bérenger, E., 2009. [95]	France	Review	Home artificial ventilation (HAV)	Duchenne muscular dystrophy; Acute anterior poliomyelitis; Obesity hypo-ventilation syndrome; Chronic obstructive pulmonary disease; Kyphoscoliosis; Dilatation of the bronchi; Apnea; Neuromuscular evolving; Tuberculosis; Various other	An overview of patients to be monitored at home, their etiology, interfaces and specific ventilators outstanding developments and benefits from technological progresses.
Egan, G.M., Siskin, G.P., Weinmann, R., Galloway, M.M., 2013. [72]	USA	Multicenter, prospective postmarket study *n* = 68 (adult patients)	Peripherally inserted central catheters (PICCs) for intravenous (IV) therapies	Active infection; Diabetes; Cancer; Human immunodeficiency virus (HIV); Cystic fibrosis	A study to evaluate the safety and efficacy of a new peripherally inserted central catheter stabilization system.
Faratro, R., Jeffries, J., Nesrallah, G.E., MacRae, J.M., 2015. [68]	Canada		Home hemodialysis (HD)	No medical diagnosis mentioned	The article outlines cannulation options for patients with arteriovenous access and describes troubleshooting techniques for potential complications; strategies are suggested to help patients overcome fear of cannulation and address problems associated with difficult cannulation.
Farrington, K., Greenwood, R., 2011. [87]	UK		Home haemodialysis	End-stage kidney failure.	An overview of developments and trends in technology for home haemodialysis.
Fayemendy, P., Sourisseau, H., Jesus, P., Desport, J.C., 2014. [58]	France	A descriptive protocol	Balloon gastrostomy feeding tubes	No medical diagnosis mentioned	The proposal of a descriptive protocol of the required equipment and the different steps of the replacement of a balloon gastrostomy feeding tube.
Feudtner, C., Villareale, N.L., Morray, B., Sharp, V., Hays, R.M., Neff, J.M., 2005. [99]	USA	Retrospective cohort study A structured hospitalization chart review *n* = 100 (patients, children)	Gastrostomy and jejeunostomy tubes; Central venous catheters; Nebulizer; Ventriculoperitoneal cerebrospinal fluid shunts; Tracheotomies	Cancer; Respiratory infections; Asthma; Gastroenteritis; Appendicitis; Epilepsy or seizures	Assessment of the proportion of children discharged from a children’s hospital who are judged to be technology-dependent, and determination of the most common devices and number of prescription medications at the time of discharge.
Fex, A., Ek, A.-C., Söderhamn, O., 2009. [25]	Sweden	Qualitative design Descriptive phenomenological methodology; Interviews *n* = 10 (patients)	Long-term oxygen therapy from a ventilator; Long-term oxygen therapy from a oxygen cylinder; Peritoneal and haemodialysis	Chronically sick patients with respiratory or kidney disorders	Description of lived experiences of self-care among persons using advanced medical technology at home.
Fex, A., Flensner, G., Ek, A.-C., Söderhamn, O., 2011a. [26]	Sweden	Qualitative design; Phenomenological hermeneutical method; Interview *n* = 10 (patients)	Long-term oxygen; Ventilator: Haemodialysis; Peritoneal dialysis	Chronically ill patients with respiratory or kidney disorders	A study to elucidate meanings of health–illness transition experiences among adult persons using advanced medical technology at home.
Fex, A., Flensner, G., Ek, A.-C., Söderhamn, O., 2011b. [42]	Sweden	Qualitative study; Hermeneutic approach; Interpretive phenomenology; Interview; Gadamerian methodology *n* = 11 (next of kin)	Long-term oxygen from a cylinder; Long-term oxygen from a ventilator; Peritoneal dialysis; Haemo dialysis	Chronic kidney or respiratory disorders	Gain a deeper understanding of the meaning of living with an adult family member using advanced medical technology at home.
Fex, A., Flensner, G., Ek, A.-C., Söderhamn, O., 2012. [43]	Sweden	Descriptive, comparative, cross-sectional, quantitative design; Questionnaire; Self-care Agency scale; Antonovsky’s sense of coherence scale *n* = 180 (patients)	Long-term oxygen; Ventilator: Haemodialysis; Peritoneal dialysis	No medical diagnosis mentioned	Report of a study of self-care agency and perceived health in a group of people using advanced medical technology at home.
François, K., Faratro, R., d’Gama, C., Wong, E., Fung, S., Chan, C.T., 2015. [69]	Canada	Single-center retrospective cohort study *n* = 84 (incident home hemodialysis patients); *n* = 56 (patients surveyed by a baseline home visit audit)	Home hemodialysis	Diabetes mellitus; Ischemic nephropathy; Glomerulonephritis; Other	A study in a university hospital-based home hemodialysis program to evaluate the effectiveness of a home visit audit tool.
Fu, M., Weick-Brady, M., Tanno, E., 2012. [14]	USA		Ventilators; Oxygen; Intravenous therapy. Invasive glucose sensor; Implantable cardioverter defibrillators; Ventricular (assist) bypass devices; Insulin infusion pumps; Piston Syringes; Automatic implantable cardioverter defibrillators with cardiac resynchronization; Peritoneal automatic delivery system; Mechanical walkers; Glucose Monitors	No medical diagnosis mentioned	The role of the US Food and Drug Administration (FDA) regarding medical devices in the home and how to support safety and safe use in the home environment.
Fung, C.H., Igodan, U., Alessi, C., Martin, J.L., Dzierzewski, J.M., Josephson, K., Kramer, B.J., 2015. [49]	USA	Descriptive study; Semi-structured in-depth interviews *n* = 19 (patients)	Positive Airway Pressure (PAP) device	Obstructive sleep apnea (OSA)	Exploration in detail of the types of difficulties experienced by patients with physical/sensory impairments who use PAP devices.
Gavish, L., Barzilay, Y., Koren, C., Stern, A., Weinrauch, L., Friedman, D.J., 2015. [34]	Israel	Prospective, randomised waiting-list-controlled trial (RCT); Document daily Numeric rating scale (NRS) pain scores; Oswestry disability index (ODI) questionnaires in a diary by participants *n* = 36 (patients)	Continuous passive motion device	Mild-to-moderate, non-specific, chronic Lower Back Pain (LBP).	Evaluation of the efficacy of a novel, angular, continuous passive motion device for self-treatment at home in patients with mild-to-moderate, non-specific, chronic low back pain.
Glader, L.J., Palfrey, J.S., 2009. [38]	USA		Nasogastric tubes; Gastronomy tubes; Indwelling venous catheters; Invasive and noninvasive mechanical ventilation	An inability to consume adequate calories to maintain reasonable nutritional status; Short bowel syndrome; Malabsorptive states; Inflammatory bowel disease: Severe dysmotility states; Other less common gastrointestinal disorders; Pneumonia; Chronic respiratory failure; Chronic lung disease; Neuromuscular disease; Central hypoventilation; Upper airway obstruction	Description of children who are dependent on technology, common indications for and complications of gastronomy tubes, invasive and noninvasive mechanical ventilation and the psychosocial effects of having a child dependent on technology.
Graf, J.M., Montagnino, B.A., Hueckel, R., McPherson, M.L., 2008. [59]	USA	Retrospective pilot case series (chart review); *n* = 70 (patients, children and adolescents)	Tracheostomies; Positive pressure ventilation	Congenital abnormalities; Neurologic diagnoses; Primary lung disease	Description of an educational program and timeline for the discharge of children with a new tracheostomy and the identification of common impediments to the education and discharge process.
Greenwald, P.W., Rutherford, A.F., Green, R.A., Giglio, J., 2004. [78]	USA	Retrospective case series (chart review) *n* = 23 (patients)	Oxygen conservers; Ventilators; Airway suction equipment	No medical diagnosis mentioned	During a widespread North American blackout, the authors identified a cluster of patients presenting to their northern Manhattan emergency department (ED) with complaints related to medical device failure. The characteristics of this group are described in an effort to better understand the resource needs of this population.
Gregoretti, C., Navalesi, P., Ghannadian, S., Carlucci, A., Pelosi, P., 2013. [85]	Italy		Mechanical ventilation	Many forms of severe chronic respiratory failure	Providing useful information to help and guide the choice of device for long-term mechanical ventilation in the home setting.
Han, Y.J., Park, J.D., Lee, B., Choi, Y.H., Suh, D.I., Lim, B.C., Chae, J.-H., 2015. [102]	South-Korea	Retrospective medical record review *n* = 57 (patients)	Home mechanical ventilation	Hereditary neuro-muscular diseases (NMDs): Spinal muscular atrophy; Congenital myopathy; Congenital muscular dystrophy; GSD type II (Pompe disease); End-stage myopathy, unspecified	Comparison of the various underlying neuromuscular diseases and an evaluation of home mechanical ventilation with regard to respiratory morbidity, the proper indications and timing for its use, and to develop a policy to improve the quality of home noninvasive ventilation.
Hanada, E., Kudou, T., 2014. [94]	Japan		Medical devices only mentioned as an example	No medical diagnosis mentioned	The paper describes the current status of ensuring electromagnetic compatibility between medical devices and wireless communications and measures against electromagnetic noise.
Heaton, J., Noyes, J., Sloper, P., Shah, R., 2005. [31]	UK	Qualitative methods; Purposive sampling strategy Face-to-face semi structured interviews; *n* = 36 (families)	Ventilators; Feeding pumps; Dialysis machines; Oxygen therapy; Intravenous drug therapies; Tracheostomies; Suction machines	Neuro-disability; Respiratory disability; Renal disability; Neuro-degenerative disability; Gastrointestinal disability; Cardiac disability; Metabolic disability; Congenital abnormality disability; Haematological disability	Families’ experiences of caring for a technology-dependent child were examined, exploring the multiple rhythms and routines around which the families’ lives were variously structured.
Hendrickson, E., Corrigan, M.L., 2013. [106]	USA	Review	Home parenteral nutrition (HPN)	No medical diagnosis mentioned	Provide nutrition support clinicians knowledge on navigating through the structured requirements of diagnosis driven billing to receive reimbursement for services related to HPN, provide information on coding, provide practical tips for surviving a Medicare billing audit, and discuss challenges of Medicare guidelines seen in clinical practice.
Hewitt-Taylor, J., 2004. [56]	UK	Descriptive study; Quantitative survey; Initial fact finding; Questionnaire *n* = 21 (staff caring for children requiring assisted ventilation)	Long-term assisted ventilation; Continuous Positive Airway Pressure (CPAP); Bilevel Positive Airway Pressure (BiPAP).	No medical diagnosis mentioned	A study of the perceived education and training needs of staff who care for children with complex needs, including assisted ventilation, and their families.
Hilbers, E.S.M., de Vries, C.G.J.C.A., Geertsma, R.E., 2013. [75]	The Nether-lands	Document analysis; Questionnaire *n* = 34 (technical documents; *n* = 18 infusion pumps; *n* = 8 ventilators; *n* = 7 dialysis systems)	Infusion pumps; Ventilators; Dialysis systems	No medical diagnosis mentioned	Investigation of the technical documentation of manufacturers on issues of safe use of their device in a home setting.
Jayanti, A., Wearden, A.J., Morris, J., Brenchley, P., Abma, I., Bayer, S., Barlow, J., Mitra, S., 2013. [55]	UK	Integrated mixed methodology; Convergent, parallel design; Quantitative methods; Qualitative study; Multicentre prospective observational cohort study Ethnographic interviews; Clinical and biomarkers; Psychosocial quantitative assessments; Neuropsychometric tests Economic evaluation; Questionnaire In-depth semi-structured interviews Groups/ study arms: a. patient b. organization c. carer d. economic evaluation 3 Patient study cohorts *n* = 500 (patients; *n* = 200 pre-dialysis; *n* = hospital haemodialysis; *n* = 100 home haemodialysis)	Home haemodialysis (HHD)	Chronic kidney disease (CKD) End stage renal disease (ESRD)	A comprehensive and systematic study of the barriers to and enablers of successful uptake and maintenance of HHD across multiple centres with low, medium and high prevalence rates of home HD. Care pathways of predialysis, incident and prevalent dialysis patients are also investigated under clinical, psychosocial and organisational domains.
Kaufman-Rivi, D., Hazlett, A.C., Hardy, M.A., Smith, J.M., Seid, H.B., 2013. [70]	USA	Descriptive study; Exploratory study; Semi-structured questionnaire for in-depth interviews and self-administration; Web-based survey adapted from semi-structured instrument Questionnaire: *n* = 22 (professional healthcare providers) Web survey: *n* = 342 (professional healthcare providers)	Negative-pressure wound therapy (NPWT) systems	No medical diagnosis mentioned	Obtain additional information about device issues that healthcare professionals face in homes settings and in extended-care facilities, as well as challenges that caregivers might encounter using this technology at home.
Kaufman, D., Weick-Brady, M., 2009. [71]	USA		There are no technologies specifically mentioned, but reference is made to complex medical devices in general. As an example are mentioned, e.g. infusion pumps, intravascular administration sets, continuous ventilators,	No medical diagnosis mentioned	The launch of the Medical Product Safety Network’s (MedSun) Subnetwork, HomeNet [a program sponsored by the U.S. Food and Drug Administration (FDA) Center for Devices and Radiological Health (CDRH)] hopes to learn about and address patient safety issues as it relates to expanding medical device usage in the home setting.
Keilty, K., Cohen, E., Ho, M., Spalding, K., Stremler, R., 2015. [39]	Canada	Systematic review; Qualitative analysis; Results presented as a narrative. *n* = 13 (studies)	Home mechanical ventilation; Non-invasive ventilation; Insulin pump therapy; Home enteral (tube) feeds; Home oxygen; Tracheostomy; Gastrostomy	Bronchopulmonary Dysplasia (BPD); Cystic fibrosis (CF); Inherited metabolic disorders (IMD); Neuromuscular (NM)	The review systematically examines studies reporting on sleep outcomes in family caregivers of technology dependent children.
Khirani, S., Louis, B., Leroux, K., Delord, V., Fauroux, B., Lofaso, F., 2013. [89]	France	Test on a lung bench with different circuit configurations and with different levels of unintentional leaks. *n* = 7 (ventilators)	Volume targeted pressure support ventilation (VT-PSV)	No medical diagnosis mentioned	Determination of the ability ofhome ventilators to maintain the preset minimal VT during unintentional leaks in a VT-PSV mode.
Kirk, S., 2010. [27]	UK	Grounded theory approach; In-depth interviews (parents were present) *n* = 28 (children/young people)	Gastrostomy/ jejunostomy; Intravenous drug therapies; Mechanical ventilation; Tracheostomy; Oxygen therapy; Parenteral nutrition; Peritoneal dialysis	No medical diagnosis mentioned	The study explores how children who need the support of medical technology for their survival and wellbeing experience and construct medical technology and its influence on their identity and social relationships.
Kirk S, Glendinning C, Callery P., 2005. [47]	UK	Grounded theory techniques; Qualitative research methods; In-depth interviews (some individual, some with both parents) *n* = 24 (children, parents of them)	Tracheostomy; Oxygen therapy; Mechanical ventilation; Intravenous drugs; Parenteral nutrition; Peritoneal dialysis; Others (e.g. gastrostomy)	Medical diagnoses mentioned in general: pre-term infants, infants with congenital impairments and children with chronic illnesses and cancer. No medical diagnoses mentioned in the study itself.	A study exploring parents’ experiences of caring for a childwho is dependent on medical technology, and in particular of performing clinical procedures on their own children.
Kropff, J., Del Favero, S., Place, J., Toffanin, C., Visentin, R., Monaro, M., Messori, M., Di Palma, F., Lanzola, G., Farret, A., Boscari, F., Galasso, S., Magni, P., Avogaro, A., Keith-Hynes, P., Kovatchev, B.P., Bruttomesso, D., Cobelli, C., DeVries, J.H., Renard, E., Magni, L., 2015. [90]	France, Italy, the Nether-lands	Multinational randomised crossover trial (open label study) *n* = 32 (patients)	Insulin pump treatment	Type I diabetes	The study assessed the effect on glucose control with use of an artificial pancreas during the evening and night plus patient-managed sensor-augmented pump therapy (SAP) during the day, versus 24 h use of patient-managed SAP only, in free-living conditions.
Lee, A.D.W., Galvao, F.H.F., Dias, M.C.G., Cruz, M.E., Marin, M., Pedrol, C.N., David, A.I., Pecora, R.A.A., Waitzberg, D.L., D'Albuquerque, L.A.C., 2014. [103]	Brazil	Patients were evaluated for a period of 6 months *n* = 128 (patients)	Home parenteral nutrition therapy (HPNT)	Intestinal failure: Mesenteric thrombosis; Colon cancer; Non-hodgkin lymphoma; Volvulus; Pseudo-obstruction; Trauma; Crohn disease; Gardner’s syndrome; Apendicitis; Peritonitis (+ dialisis); Provoked abortion	The article profiles a Brazilian single-center experience with 128 cases of HTPN followed for the last 30 years and appraise the referral for potential intestinal and multivisceral transplantation.
Leger, S.S., 2005. [84]	France	Review	Mechanical ventilation	Chronical diseases	The article aims to examine the different indications of a humidification system in patients with mechanical ventilation in the home, to review the literature in order to identify the positive results obtained by humidification and, finally, to describe the most efficient types of humidifiers.
Lehoux, P., 2004. [48]	Canada	Qualitative study, relied on the triangulation of three sources of data: 1) interviews with patients (*n* = 16); 2) interviews with carers (*n* = 6); 3) direct observation of nursing visits of a different set of patients (*n* = 16).	Intravenous therapy; Parenteral nutrition; Peritoneal dialysis; Oxygen therapy	No medical diagnosis mentioned	Documentation, from the patient’s perspective, of how the level of user-friendliness of medical technology influences its integration into the private and social lives of patients. Understanding what makes a technology user-friendly should help improve the design of home care services.
Lehoux, P., Charland, C., Richard, L., Pineault, R., St-Arnaud, J., 2002. [5]	Canada	Postal questionnaire *n* = 97 (local centers)	Intravenous pump therapy; Oxygen therapy; Peritoneal dialysis; Haemo dialysis; Parenteral nutrition;	No medical diagnosis mentioned	The article describes various medical technologies that are used frequently in the home and the responsibility of local community service centers in the region of Quebec, Canada.
Lehoux, P., Saint-Arnaud, J., Richard, L., 2004. [30]	Canada	Biographical interview, interview questionnaire; Direct observations; Document analysis (patient manuals, brochures, leaflets) *n* = 16 (patients) *n* = 6 (caregivers) *n* = 16 (home visits by nurses) *n* = 26 (documents)	Intravenous therapy, Parenteral nutrition, Peritoneal dialysis; Oxygen therapy	Patients with recurring infections; Chronic obstructive pulmonary disease; Renal failure	Determination of how specialised medical equipment by patients at home was supposed to be used versus how it was actually used.
Lemke, M.R., Mendonca, R.J., 2013. [50]	USA		Dialysis; Intravenous therapies	No medical diagnosis mentioned	The article describes several aspects of accessibility of medical devices for home healthcare recipients, especially lay users.
Lewarski, J.S., Gay, P.C., 2007. [22]	USA		Home mechanical ventilation	Medical diagnoses only mentioned as an example.	The article explains several issues in home mechanical ventilation, such as policies and practice standards, costs, reimbursement and coverage
Matsui, K., Kataoka, A., Yamamoto, A., Tanoue, K., Kurosawa, K., Shibasaki, J., Ohyama, M., Aida, N., 2014. [98]	Japan	Clinical data review/ charts review *n* = 10 (patients)	Suction apparatus; Tube feeding; Gastrostomy; Tracheostomy; Oxygen therapy; Ventilator	Möbius syndrome	Investigation of the outcome of patients with Möbius syndrome, including the mortality rate, rate of neonatal intensive care unit (NICU) admission, neurological findings, developmental problems, and medical home care and device needs.
McGoldrick, M., 2010. [67]	USA	Article presents evidence based guidelines and recommendations on the preferred methods.	Oxygen concentrators, Ventilators; Continuous positive airway pressure (CPAP); Bilevel positive airway pressure (BiPAP); Nasal cannulas; Tracheostomy tubes; Tracheal suction catheter; Nebulizers	An immune-compromised individual with a chronic underlying illness	This article presents evidenced based guidelines and recommendations on the preferred methods for managing respiratory equipment and supplies commonly used by patients in the home setting and conducting surveillance activitiesto ultimately prevent respiratoryinfections.
Michihata, N., Matsui, H., Fushimi, K., Yasunaga, H., 2015. [101]	Japan	Database analysis (The Japanese Diagnosis Procedure Combination (DPC) database) *n* = 4729 (patients)	Tracheostomy tube; Gastrostomy tube; Home respirator; Home centralvenous alimentation	Chromosomal anomaly; Malignancy; Inborn error of metabolism (IEM); Congenital heart disease (CHD); Immune deficiency; Endocrine diseases; Cerebral palsy; Other congenital anomalies; Epilepsy; Other diseases Ischemic heart diseases, including angina pectoris; Acute myocardial infarction; Cerebrovascular diseases; Lung, gastric, colon, hepatic, breast, uterus, and prostate cancer	Determination of the clinical details of adult patients admitted to pediatric wards in Japanese acute-care hospitals.
Munck, B., Fridlund, B., Mårtensson, J., 2011. [53]	Sweden	Descriptive design; Phenomenographic approach; Qualitative study; Semi-structured interview *n* = 16 (nurses)	There are no technologies specifically mentioned in the study itself, but reference is made to complex medical devices according to a definition and examples. ‘Medical technology was defined and confined to the more advanced devices that may be present in the home, such as ventilators, suction devices, oxygen and various ports and pumps’.	No medical diagnosis mentioned	Description of district nurses’ conceptions of medical technology in palliative homecare.
Munck, B., Sandgren, A., Fridlund, B., Mårtensson, J., 2012a. [36]	Sweden	Explorative descriptive design; Phenomenographic approach; Qualitative study Semi-structured interview *n* = 15 (next-of-kin)	Pain, nutrition and volume pumps; Oxygen concentrators; Suctions and inhalation devices; Percutaneous endoscopic gastronomy (PEG); Subcutaneous vein ports.	No medical diagnosis mentioned	Description of next-of-kin’s conceptions of medical technology in palliative homecare.
Munck, B., Sandgren, A., Fridlund, B., Mårtensson, J., 2012b. [52]	Sweden	Qualitative analysis; Explorative descriptive design; Phenomenographic approach; Interview *n* = 15 (patients)	Pain pumps; Nutrition and volume pumps; Intravenous infusion: Disetronic pen for subcutaneous injections; Oxygen concentrators and cylinders; Nephrostomy catheters; Percutaneous endoscopic gastronomy; Subcutaneous venous port implantation.	Different types of cancer; Amyotrophic lateral sclerosis (ALS); Heart failure; Chronic obstructive disease.	Description of the patients’ ways of understanding medical technology in palliative home care.
Nakayama, T., Tanaka, S., Uematsu, M., Kikuchi, A., Hino-Fukuyo, N., Morimoto, T., Sakamoto, O., Tsuchiya, S., Kure, S., 2014. [76]	Japan	Retrospective study; Medical records were hand-reviewed to identify inpatients Survey by questionnaire *n* = 24 (patients)	Ventilator; Peritoneal dialysis; Oxygen condenser	Neurological disorders: Periparturient disorder; Mitochondrial disease; Congenital myopathy; Epilepsy; Cerebral sequelae of acute encephalopathy; Perizeus Merzback disease Kidney disorders: Hypoplastic kidney; Nephrotic syndrome Others: Diabetes mellitus type 1; Long QT syndrome; Effects from bone marrow transplantation, chronic respiratory failure	Effect of a blackout in pediatric patients with home medicaldevices during the 2011 eastern Japan earthquake
Paddeu, E.M., Giganti, F., Piumelli, R., De Masi, S., Filippi. L., Viggiano, M.P., Donzelli, G., 2015. [40]	Italy	Pittsburgh Sleep Quality Index (PSQI) questionnaire; Epworth Sleepiness Scale (ESS); Beck Depression Inventory (BDI-II); Beck Anxiety Inventory (BAI) *n* = 23 (parents of 23 children with CCHS) *n* = 23 (parents of 23 healthy children)	Mechanical ventilation (via nasal mask or tracheostomy)	Congenital central hypoventilation syndrome (CCHS)	The daily challenges associatedwith caring for technology-dependent children can place primary caregivers under significant stress, especially at night. The study investigated how this condition affects mothers and fathers by producing poor sleep quality, high-level diurnal sleepiness, anxiety, and depression.
Paul, J., Otvos, T., 2006. [82]	Canada	Randomized crossover study; Measurement by oximeter; Questionnaire *n* = 25 (patients)	Oxygen therapy	Ex-smokers with severe chronic obstructive pulmonary disease	Comparison of the performance of a new oxygen delivery device, the OxyArm (OA) (Southmedic Inc., Canada), with a standard nasal cannula (NC) (Salter-Style 1600, Salter Labs, USA) for both oxygen delivery and patient preference in patients on long-term oxygen therapy (LTOT).
Pourrat, M., Neuville, S., 2007. [73]	France	Survey; questionnaire *n* = 12 (by law authorized centres) *n* = 6 (service providers) *n* = 0 (custom-made makers) *n* = 0 (laboratories)	Home parenteral nutrition	No medical diagnosis mentioned	For Home Parenteral Nutrition (HPN), pharmacy had to deliver some medical devices and drugs. It comes up the following and taking care of incidents that’s occurring at home with those products. The article describes an inventory on vigilance’s organization, incident’smanagement and assessment, about HPN in France.
Pourtier, J., 2013. [97]	France		Patient-controlled analgesia pumps.	No medical diagnosis mentioned	Technology for improving pain management in the home; various aspects related to analgesia pumps.
Prenton, S., Kenney, L.P., Stapleton, C., Cooper, G., Reeves, M.L., Heller, B.W., Sobuh, M., Barker, A.T., Healey, J., Good, T.R., Thies, S.B., Howard, D., Williamson, T., 2014. [92]	UK	Feasibility study Purposive questionnaires Paper diary *n* = 7 (patients)	Functional electrical stimulation system	Unilateral foot-drop of central neurologic origin (>6mo)	Investigation of the feasibility of unsupervised community use of an array-based automated setup functional electrical stimulator for current foot-drop functional electrical stimulation (FES) users.
Rajkomar, A., Farrington, K., Mayer, A., Walker, D., Blandford, A., 2014. [51]	UK	Qualitative method Ethnographic observations; Semi-structured Interviews *n* = 19 (patients and their carers)	Home haemodialysis technology	No medical diagnosis mentioned	An inventory of patients’ and carers’ experiences of interacting with home haemodialysis (HHD) technology, in terms of user experience, how the design of the technology supports safety and fits with home use, and how the broader context of service provision impacts on patients’ use of the technology.
Rajkomar, A., Mayer, A., Blandford, A., 2015. [79]	UK	Ethnographic observations; Semi-structured interviews; Distributed cognition for teamwork methodology	Home hemodialysis technology (HHT)	Renal patients / kidney failure	In this study, Distributed Cognition (Dcog) was applied to understand renal patients’ interactions with Home Hemodialysis Technology (HHT), as an example of a home medical device.
Rand, D.A., Mener, D.J., Lerner, E.B., DeRobertis, N., 2005. [77]	USA	Retrospective case series (medical record review) *n* = 83 (medical records)	Home respiratory equipment; Home nebulizers; Oxygen devices	No medical diagnosis mentioned	Description of the experience of an urban, commercial ambulance provider during the multistate August 2003 electrical power outage (EPO) and to identify how such an event can affect an emergency medical services (EMS) system.
Scala, R., 2004. [88]	Italy	*n* = 29 (devices)	Bi-level home ventilators for non invasive positive pressure ventilation	Chronic respiratory failure (due to neuro-muscular disorders); COPD; Severe chest wall deformity; Obesity	The author describes the technical aspects, the individual characteristics and the clinical applications of the most common used bi-level ventilators.
Short, D., Norwood, J., 2003. [108]	UK	Phase 1: Survey (Semi-structured interview); Phase 2: Case study analyses (in-depth case study analyses of selected districts) *n* = 98 (health authorities)	Parenteral nutrition; Intravenous antibiotics; Intravenous chemotherapy; Continuous ambulatory peritoneal dialysis	Cystic fibrosis; Cancer	The study addresses questions: Why is high-tech healthcare at home purchasing underdeveloped and what could be done to improve it
Siewers, V., Holmøy, T., Frich, J.C., 2013. [54]	Norway	Qualitative study; Semi-structured in-depth interviews *n* = 5 (patients)	Mechanical insufflation – exsufflation (MI-E)	Amyotrophic lateral sclerosis (ALS)	The study explores patients’, family carers’ and health professionals’ experiences with using mechanical insufflation – exsufflation (MI-E) in amyotrophic lateral sclerosis (ALS) in the home setting.
Southey, D., Pullinger, D., Loggos, S., Kumari, N., Lengyel, E., Morgan, I., Yiu, P., Nandi, J., Luckraz, H., 2015. [105]	UK	Observational study; Data collected prospectively on the thoracic database; Data logged in a specific data sheet *n* = 20 (patients)	Portable digital suction device	‘All patients who underwent a thoracic procedure and who required suction postoperatively for a persistent air leak and a confirmed air-space within the pleural cavity’	Patients undergoing thoracic surgical procedures who met strict discharge criteria were allowed to continue their treatment at home with the device. They were monitored in a designated follow-up clinic. Data were collected to identify the impact of this service in relation to the duration of follow-up required, bed-days saved, and potential cost/benefits.
Stieglitz, S., George, S., Priegnitz, C., Hagmeyer, L., Randerath, W., 2013. [66]	Germany	Case series *n* = 3 (patients)	Invasive and non-invasive ventilators	COPD; Lung cancer; Chronic ventilator failure as a consequence of chronic obstructive pulmonary disease	The article describes life-threatening events in respiratory medicine: misconnections of invasive and non-invasive ventilators and Interfaces
Su, C.-L., Lee, C.-N., Chen, H.-C., Feng, L.-P., Lin, H.-W., Chiang, L.-L., 2014. [81]	Taiwan	Retrospective, cross-sectional, observational survey design; Questionnaires; Walking test (patient self score) *n* = 42 (patients using LOG) *n* = 102 (patients using OCG)	Long-term oxygen therapy	Chronic respiratory insufficiency; Chronic obstructive pulmonary disease (COPD); Restrictive lung disease; Neuromuscular diseases; Cancer; Interstitial lung diseases	The study compared oxygen usage between patients from a liquid oxygen group (LOG) and an oxygen concentrator group (OCG). The authors also assessed the physiologic responses of patients with chronic obstructive pulmonary disease (COPD) to ambulatory oxygen use at home.
Sunwoo, B.Y., Mulholland, M., Rosen, I.M., Wolfe, L.F., 2014. [57]	USA		Home noninvasive ventilation technology	Neuromuscular disease (including amyotrophic lateral sclerosis and Duchenne muscular dystrophy); Scoliosis; Restrictive chest wall disease; Restrictive thoracic disorders; COPD/severe COPD; The overlap syndrome or coexisting COPD and OSA; Sleep-related breathing disorders; Central or complex sleep apnea; Obesity hypoventilation syndrome (OHS); Hypoventilation syndromes	The article provides a practice management perspective for clinicians providing home noninvasive ventilation, including coverage, coding, and reimbursement to optimize clinical care and minimize lost revenue.
Szeinbach, S.L., Pauline, J., Villa, K.F., Commerford, S.R., Collins, A., Seoane-Vazquez, E., 2015. [65]	USA	Retrospective chart review Qualitative study (the interview part) One-on-one interviews *n* = 163 (patients)	Home parenteral nutrition	Intestinal obstruction; Acute pancreatitis; Hyperemesis metabolism; Regional enteritis; Intestinal disorders, ulceration; Intestinal malabsorption; Enterocolitis; Sepsis; Stomach ulceration with perforation; Acute intestinal vascular insufficiency; Intestinal fistula; Gastroparesis; Persistent vomiting, pneumonitis; Other gastrointestinal issues, disturbances; Oncology-related diagnoses	The article describes catheter complications and outcomes in patients who received home parenteral nutrition (HPN) therapy.
Tanno, E., 2010. [74]	USA	*n* = 6 (hospitals)	There are no technologies specifically mentioned in the study itself, but reference is made to complex medical devices as an example.	No medical diagnosis mentioned	Because patients, who use home medical technologies, are so dependent on these devices they bring them into hospitals when they seek treatment. Many hospitals have developed specific protocols, including safety inspections by clinical engineers, to follow when a home-use device is brought in. This article summarizes the policies that 6 hospitals have developed to address this situation.
Tearl, D.K., Cox, T.J., Hertzog, J.H., 2006. [61]	USA	Demographic data are prospectively collected from databases; Surveys conducted over the telephone or via facsimile *n* = 74 (patients)	Respiratory technology; Ventilator; Continuous positive airway pressure (CPAP); Tracheostomy collar; Negative-pressure ventilator (NVP); Bi-level positive airway pressure (BiPAP)	Respiratory failure: Airway obstruction; Neuromuscular/ Spinal-cord injury (SCI); Bronchopulmonary dysplasia (BPD)	Preparation of respiratory-technology-dependent children for hospital discharge presents many challenges. Adequate training and education of parental caregivers, discharge planning, and coordination with the durable-medical-equipment and home-nursing companies must be completed. The role of a dedicated Respiratory care discharge coordinator has been evaluated in this study.
Tennankore, K.K., D’Gama, C., Faratro, R., Fung, S., Wong, E., Chan, C.T., 2014. [80]	Canada	Retrospective cohort study (all characteristics collected based on identification in electronic records and patient charts) *n* = 202 (patients)	Home hemodialysis	End-stage renal disease: Diabetes; Glomerulonephritis; Polycystic kidney disease	The study describes adverse technical events in a large cohort of home hemodialysis patients.
Thomson, R., Martin, J.L., Sharples, S., 2013. [28]	UK	Qualitative study; In-depth semi-structured interview *n* = 12 (patients)	Transcutaneous electrical nerve stimulation device; Oxygen concentrator; Continuous ambulatory peritoneal dialysis; Stair-lift; Nebulizer	Diabetes	The article describes the psychosocial impact of home use medical devices on the lives of older people and how the devices are integrated into their lives.
Toly, V.B., Musil, C.M., Carl, J.C., 2012. [37]	USA	Descriptive, correlational, dross-sectional study; Structured interview, face-to-face, using the Demographic Characteristics Questionnaire, the Functional Status II–Revised Scale, the Center for Epidemiological Studies–Depression Scale, a Normalization Scale subscale, and the Feetham Family Functioning Survey. *n* = 103 (mothers)	Mechanical ventilation; Intravenous nutrition/ medication; Respiratory/ nutritional support; Apnea monitors; Feeding tube; Tracheostomy tube; Supplemental oxygen	Neuromuscular; Respiratory conditions; Gastrointestinal conditions; Cardiac conditions; Cystic fybrosis; Metabolic disorders; Renal disorders	The study describes various issues related to family functioning and normalization in mothers of children dependent on medical technology following initiation of home care.
Toly, V.B., Musil, C.M., Zauszniewski, J.A., 2014. [41]	USA	Longitudinal randomized controlled pilot trial; Structured interviews; Semi-structured exit Interviews *n* = 22 (mothers)	Mechanical ventilation; Intravenous nutrition/ medication; Respiratory/ nutritional support.	Respondents recruited from pulmonology and gastroenterology clinics	The purpose of the study was to determine the feasibility, acceptability, and efficacy ofresourcefulness training (RT), a cognitive–behavioral intervention, among mothers of technology-dependent children.
Wang, K.-W.K., Barnard, A., 2004. [35]	Australia	Empirical review	Mechanical ventilation; Tracheostomy; Oxygen therapy; Enteral nutrition; Parenteral nutrition; Intravenous drug therapies; Peritoneal dialysis; Haemodialysis; Suction devices	No medical diagnoses mentioned, only as an example.	The paper provides a comprehensive literature review on caring for technology-dependent children living at home to gain an understanding of the development of paediatric home care, and its impact on technology-dependent children and their families, and social implications.
Weiler-Ravell, D., 2002. [107]	Israel		Respiratory support, ventilators	Neuromuscular respiratory failure Chronic obstructive pulmonary disease	The article describes the quandary of home-care respiratory management.
Wong, J., Eakin, J., Migram, P., Cafazzo, J.A., Halifax, N.V.D., Chan, C.T., 2009. [60]	Canada	Qualitative study; Semi-structured interviews; Focusgroup *n* = 23 (patients; 15 interviews; 8 focus group)	Home hemodialysis	End stage renal disease (ESRD).	The study explores patient training experiences with learning a complex medical device for the selfadministration of nocturnal hemodialysis at home.
Yik, Y.I., Ismail, K.A., Hutson, J.M., Southwell, B.R., 2012. [91]	Australia	Prospective study; Bowel diaries; Questionnaires; Colonic transit studies *n* = 32 (patients)	Transcutaneous electrical stimulation	Slow-transit constipation (STC)	The article describes the test of the effectiveness of home transcutaneous electrical stimulation (TES) when patients with slow-transit constipation (STC) were trained by a naive clinician.

### Appraisal of selected studies

To conduct the systematic literature search on the international state of art concerning various aspects of the use of advanced medical technologies at home, several sources are consulted. To guarantee a scientific standard, only articles were retrieved from academic databases. MEDLINE refers to journals for biomedical literature from around the world; Cinahl contains an index of nursing and research journals covering nursing, biomedicine, health sciences librarianship, alternative medicine, allied health and more. These databases related to discipline have been supplemented with Scopus, which is considered to be the largest abstract and citation database of peer-reviewed literature. Grey literature, such as national and international reports on regulations and safety of medical technologies, is also used to illustrate the background of the problem statement and describe definitions. The Classification of advanced medical technologies in the Netherlands according to the National Institute for Public Health and the Environment (RIVM) has been used as a framework to categorise the medical technologies in the selected articles. No methodological conditions of selected studies were applied in advance and the quality criterion we applied was that of the article had to contain empirical material, as we wanted to obtain an comprehensive overview of published studies of any design and to get insight in a variety of contents.

## Results

### Categorization of included articles

The characteristics of the included articles are outlined in Table 3. All included articles were categorized by year of publication and the type of research, like the designs, methods and used instruments in the studies. Research features were synthesized where possible into overarching categories. For example, ‘systematic review’ and ‘narrative review’ were scored as ‘review’ and instruments as ‘semi-structured interview’ and ‘in-depth individual interview’ were both assigned to the category ‘interview’.

For each study, the medical technology or technologies on which the study was based was scored. The categorization was in accordance with the classification of AMTs (see Table 1). For example, the devices ‘continuous positive airway pressure (CPAP)’ and ‘negative pressure ventilation (NPV) have both been categorized as ‘respiratory support’; and the devices ‘jejeunostomy tube’ and ‘gastronomy tube’ as ‘enteral nutrition’. With regard to the category ‘dialysis’, further subdivision was made by using ‘haemo dialysis’ and ‘peritoneal dialysis’. If in an article a medical technology was mentioned as an example, but was no subject of study, then the technology was not scored.

‘Medical diagnosis (or diagnoses)’ as mentioned in the studies, was included in the analysis only if it was related to the medical technology as the subject of study, not if it has been mentioned as an example. In some cases, an underlying cause of diagnosis was indicated. For example, ‘chronic respiratory failure due to congenital myopathy’, in itself a neurological disorder, has been scored as ‘neurological disorder’. Diseases or disorders have been classified as much as possible under the overarching name. For example ‘pneumonia’ and ‘cystic fibrosis’ are categorized under ‘respiratory failure’, and ‘gastroparesis’ and ‘Crohns disease’ under ‘gastrointestinal disorder’. The category ‘other’ contains diagnoses which occur only once, such as ‘chromosomal anomaly’, or which are not yet determined, like ‘chronic diseases’ or ‘congenital abnormalities’.

In relation to the research questions, articles were classified regarding one of the following categories and, where appropriate, into subcategories:

1. User experiences
2. Training, instruction and education
3. Safety, risks, incidents and complications

From an analysis of the articles, additional categories of content emerged:

4. Design and technological development
5. Application with regard to certain diseases or disorders, indication for and extent of use
6. Policy and management

### Types of medical technologies used, frequency of use and trends

In four of the 87 articles (5%) there were no specific medical technologies mentioned as a subject of study (see Table 4). Almost half of the studies (45%) considered medical technologies for respiratory support and 39% devices for dialysis, either haemo- (*n* = 18), peritoneal- (*n* = 15) or dialysis not specified (*n* = 1). Of the studies, 29% reported on devices for oxygen therapy. In addition, there has been relatively more research conducted on equipment for ‘infusion therapy’ (*n* = 19; 22%), parenteral nutrition and enteral nutrition with a score of 20% each (*n* = 17). Relatively little research has been carried out on suction devices (8%), external electrostimulation (5%), nebulizer (5%), insulin pump therapy (3%), sleep apnea treatment (2%), patient lifting hoists (2%), vacuum assisted wound closure (1%) and continuous passive motion (1%). None of de studies considered medical technologies with regard to decubitus treatment, skeletal traction or UV (ultraviolet) therapy.

**Table 4:**
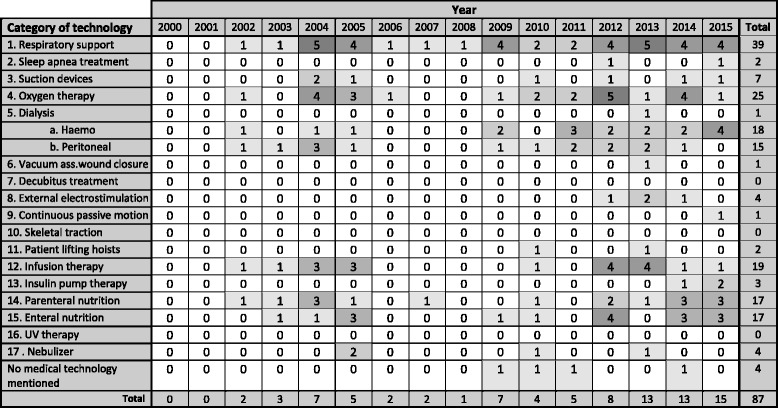
Trends in papers reporting on AMTs (n = 87, multiple answers possible), by year of publication (2000–2015)

No shading *n* = 0, up till the darkest shading *n* = 5

Table 4 shows that on the years 2000 and 2001 no relevant articles on the subject were found. Over the period 2000–2005, 17 articles were published, the same number over 2006–2010, and there has been a substantial increase in the number of publications to 54 over the years 2011–2015. In general, it can be concluded that more frequent investigated technologies show a fairly even distribution of publications over the years 2000–2015. Technologies, on which little research had been done, except for nebulizers, have been mainly investigated since 2010. An increase of published articles over the years 2000–2015 is apparent particularly for haemo dialysis and to a lesser extent, for devices for enteral- and parenteral nutrition. As mentioned before, several studies reported on the increase of the number of medical technologies used in home settings, but concrete data are not available. However, the number of studies and the visible trends may be indicative of the frequency of use.

**Fig. 2:**
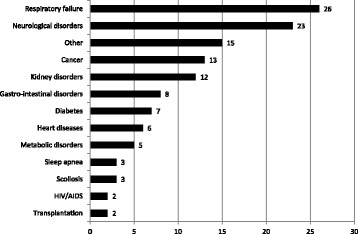
Number of medical diagnoses mentioned in articles on AMTs (*n* = 87, multiple answers possible)

In 63% of the cases (*n* = 55), a medical diagnosis (or diagnoses) was mentioned in the article. Where a diagnosis has been mentioned, in almost half of the studies (*n* = 26; 47%) it concerned diagnoses in the field of respiratory failure (see Fig. 2). This is not surprising, since ‘respiratory support’ is the medical technology most commonly found in the articles, similarly ‘oxygen therapy’ has also been considered relatively often. Diagnoses with regard to neurological disorders occurred in 42% of the studies (*n* = 23). Just over a quarter of the studies (27%) considered diagnoses ‘other’, such as ‘sepsis’, ‘chromosomal anomaly’ or other not specified medical disorders, nearly a quarter (24%) considered ‘cancer’ and 22% kidney disorders (*n =* 12).

An analysis of the used research designs identified that 64% (*n* = 56) of the studies used an observational (non-experimental) design and only a small part of the studies (*n* = 5; 6%) used an experimental design, such as a Randomized Control Trial (RCT). Of the included studies 19 were reviews and 8 were essays. A quantitative design (*n* = 37) was used more frequently than a qualitative design (*n* = 25); and only one study applied ‘mixed methods’ (quantitative and qualitative). Just over one-third of the studies (35%) used a descriptive design, and a similar number used a cross-sectional study (36%). Case series were used in 12% of the articles and a cohort-study in 9%. A phenomenological approach was applied in 16% of the records. Research instruments most frequently used were interviews (33%) and survey/questionnaires (21%). In 10% of the cases other instruments were used, including different types of assessments or tests.

**Fig. 3:**
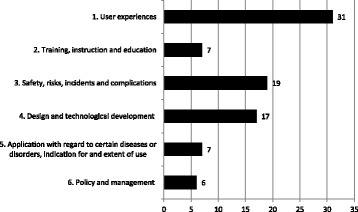
Number of articles on AMTs with main content categories (*n* = 87)

With regard to the categories of content, most research has been carried out on ‘user experiences’ (see Fig. 3): just over one-third of the articles (*n* = 31; 36%) focused on this topic. Of these articles almost all studies focused on experiences of patients or informal caregivers (*n* = 29) and only a small number (*n* = 2) considered the user experiences of nurses or other professionals (see Table 5). More than half of the studies (*n* = 19) used a qualitative research design; of these 13 used a phenomenological approach. The goal of these studies was to elicit the essence of human phenomena as experienced by the users. Seven studies used a quantitative design and one an integrated mixed method. Three of the studies applied a grounded theory approach and two an experimental design (randomized controlled trial). The research instruments in this content category to collect data were interviews, either semi-structured or in-depth, and a survey. About two-thirds of the articles regarding ‘user experiences’ were published in the period 2011–2015, with an accent on the psychosocial impact of patients or informal caregivers.

**Table 5:**
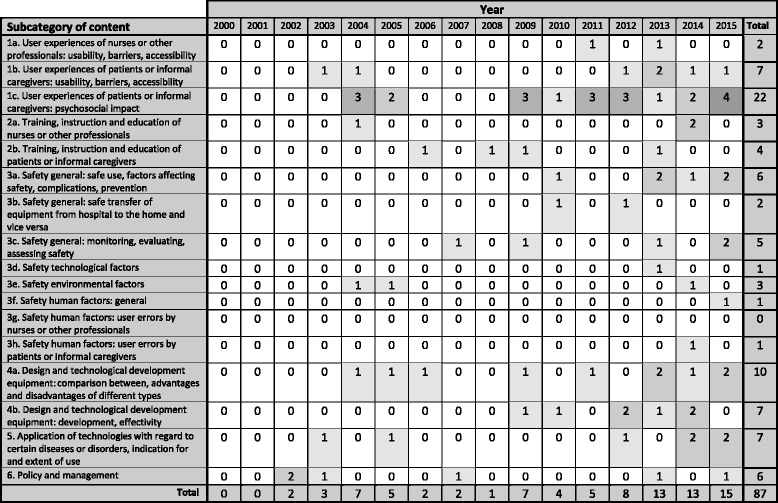
Subcateogories of content in selected articles on AMTs (*n* = 87) by year of publication (2000–2015)

No shading *n* = 0, up till the darkest shading *n* = 4

Relatively little research was found on ‘training, instruction, education’ (*n* = 7), for the use of AMTs in home settings. It was remarkable that all the studies identified as focusing on this topic, concentrated on one category of AMT. Respiratory support was the subject of study in four instances and in the other three, the focus was on technologies for enteral nutrition, haemo dialysis and external electro-stimulation. Four of the seven articles utilized quantitative methods, among which three of them used an observational non-experimental design and one was an experimental randomized double-blind clinical trial. Another study within the initial seven articles used a qualitative observational non-experimental design, one was a review and another was in essay format.

In total, 22% of the articles discussed topics on safety, risks, incidents and complications (*n* = 19). In the majority of cases (*n* = 13) general aspects about the subject, for instance safe use, factors affecting safety, a safe transfer of the equipment and monitoring of assessing safety were considered. One article described technological factors with regard to safety, three articles reported on environmental factors and two explored human factors. Safety aspects were explored over a wide range of medical technologies. Five articles were reviews and one an essay. Quantitative methods were used in ten of the cases, particularly for monitoring, evaluating and assessing safety, technological and environmental factors. Only three studies used a qualitative design. Retrospective chart reviews or case series were used to collect data in some cases of unforeseen events. Table 5 shows about a doubling of published articles in the period 2011–2015 regarding this content category, compared to the previous period 2000–2010.

Approximately 20% of the selected articles considered the content category ‘design and technological development of the medical device’ (*n* = 17). The studies each focused on only one type of AMT and treated a relative wide range of eight different categories, such as ‘respiratory support’, ‘oxygen therapy’, ‘haemo dialysis’, ‘infusion therapy’, ‘insulin pump therapy’ and ‘enteral nutrition’, but also ‘external electrostimulation’ and ‘patient lifting hoists’. Interestingly, in this group of articles, relatively often (*n* = 6) no medical diagnosis was mentioned. Around half of the studies (*n* = 8) referring to this topic were in review or essay format. All other studies used a quantitative research design and throughout the search no application of qualitative designs were found. Two studies used an experimental study design (randomized crossover trial) to obtain data and two described a prospective cohort study. The majority of papers (*n* = 11) were published in the period 2011–2015 and six in the preceding period up to and including 2010.

Seven articles concerned the application of AMTs, all of them devices with regard to at least respiratory support and/or nutritional support. Five studies used a non-experimental quantitative design including the analysis of clinical data, such as record reviews or cohort studies, and two articles were reviews. Most articles on this subject (*n* = 5) were published in the period 2012–2015.

Six articles described policy or management systems in different countries regarding the use of AMTs at home. The majority of the articles (*n = 4*) were in essay or review format. The other papers concerned a qualitative cross-sectional case study analysis and an observational quantitative study in which data are collected prospectively using a database. The categories of content will now be discussed in greater detail.

### Content description and trends to secondary research questions

#### User experiences

In this category, 22 articles described the psychosocial impact on patients or informal caregivers from the use of medical technologies at home. Living at home with the assistance of medical technology needs a range of adjustments. Fex et al. [25, 26] state that self-care is more than mastering the technology, in terms of the health-illness transition, it requires ‘…. an active learning process of accepting, managing, adjusting and improving technology’. When it comes to children, they have to learn to incorporate disability, illness and technology actively within their process of growing up [27]. It seems that the use of medical technologies in the home can have both a positive and a negative psychosocial impact on patients and their families, which in turn causes ambivalence in experiences [27, 28]. On the one hand, patients in general gain more independence, an enhanced overall health and a better quality of life [29–34]. On the other hand, for some patients the experience is one of dependency on others for executing daily activities, and these circumstances, to some extent, a social restricted live and perceived stigmatization [29, 30]. The situation in which patients need to use medical technology at home also affects family functioning and requires next of kin responsibilities [35–37]. As a result, next of kin caregivers are frequently faced with poor sleep quality and quantity, and/−or other significant psychosocial effects [38–41]. Nevertheless, family members had a positive attitude to the concept of bringing the technology into the home [42]. Knowledge of how to use the technology and permanent access to support from healthcare professionals and significant others, enabled next of kin caregivers to take responsibility for providing necessary care and to facilitate patients learning to provide self-care [25, 36, 42–44]. Bezruczko et al. [45, 46] developed a measure of mothers’ confidence to care for children assisted with medical technologies in their homes. To provide high quality sustainable care, nurses have to recognize and understand the psychosocial dimensions for both patients and family members which arise as a result of changing role and providing care for the patients. The need to provide emotional support and support with appropriate coping strategies is a key professional role [25, 26, 47]. Insight into the psychosocial effects on those involved can be used to assist designers of medical devices to find strategies to better facilitate the integration of these technologies into the home [28].

Seven articles reported on the usability, barriers and accessibility experienced by patients or informal caregivers. Findings in these studies showed that several technologies were rarely perceived as user-friendly and that home medical devices inadequately met the needs of individuals with physical or sensory deficits [48, 49]. An accessible design which meets the diversity of individual user needs, characteristics and features would be better able to help patients manage their own treatment and so could contribute to the quality of care and safety of patients and lay users [50, 51]. Munck et al. [52] stated that restricted patients were reminded daily of the medical technology and were more dependent on assistance from healthcare professionals than masterful patients.

In contrast to the group of patients or informal caregivers, only two papers in this content category focused on the user experiences of nurses or other professional caregivers. The review demonstrates that to maintain patient safety, more education on application of medical devices for users is needed together with improved awareness and understanding of how to use the medical technology correctly in a patient-safe way [53, 54]. More collaboration between all involved ‘actors’ in the process of care is also requisite. Continuity among carers, trust between patient and carers and supportive communication between informal and professional caregivers are important factors for the successful implementation of medical technologies in the home environment while maintaining patient safety [44, 51, 53–55].

#### Training, instruction and education

Three articles regarding this topic focused on nurses or other professionals and four on the patients or informal caregivers. The results showed that successful use of advanced medical technologies at home requires adequate staff education and training programmes. Although many topics in educational programmes are suitable for different types of professionals in care provision, the focus for the level and application of information can vary for Registered Nurses and unregistered care staff. In addition, for overall learning experiences to be of maximum benefit there is a need for a clear focus on the specific client groups [56]. According to Sunwoo et al. [57], in the case of home non-invasive ventilation the degree of clinical support needed is extremely variable given the mixed indications for this respiratory support. A relatively simple procedure, such as the replacement of a feeding tube, can be performed by nurses, the patient and informal caregivers, provided they are trained well [58]. However, several studies revealed the complexity of the education needed by patients and informal caregivers for the use of advanced medical technologies at home [59, 60]. Nevertheless, the studies revealed that a structured education programme, specific training, or the support of a dedicated discharge coordinator has several advantages [59, 61, 62]. It was evident that good preparation by patients or informal caregivers may result in a shorter length of stay in hospital, a better performance with regard to the use of the equipment or less requests by patients and/or families for assistance.

#### Safety, risks, incidents and complications

Most articles regarding this topic (*n* = 13) reported on safety in general, like aspects of safe use, factors affecting safety, complications and prevention of incidents in the home. Some identified the risk factors and the complications that may arise [63–65], where Stieglitz et al. [66] also emphasize that human error is the main reason for critical incidents and that regular instruction for medical staff and patients is necessary. To prevent untoward and adverse events, evidence based guidelines, recommendations on the preferred methods for managing the equipment, troubleshooting techniques for potential complications and monitoring activities are necessary [67, 68]. Faratro et al. [68] added that key performance and quality indicators are important mechanisms to ensure patient safety when using a medical device in the home. Methods to address or evaluate patient safety issues are for example, a home visit audit tool, a nationwide adverse event reporting system, programs such as the Medical Product Safety Network HomeNet, or, in the case of peripherally inserted central catheters (PICCs) a central catheter stabilization system [69–72]. However, a study conducted by Pourrat and Neuville [73] in France found that there are very few internal medical devices vigilance reports found within organizations that deliver devices for home parenteral nutrition and that safety management could be improved. The safe transfer of medical devices from a hospital setting to the home and vice versa, comes with several challenges regarding technological, environmental and human factors [14]. While many hospitals have developed policies to control the pathways of home-used devices in the hospitals, in case patients take them into the hospital when they are admitted for treatment [74]. Improvement of the safety of devices intended for use in home settings, implies also improvement of safety when their transfer to the hospital settings is urgently needed.

One article considered the technological factors, three the environmental and two the human factors. An example of research on the technological factors of safety related aspects of medical technologies used in home settings by Hilbers et al. [75] found that manufacturers pay insufficient attention to safety-related items in technical documentation for the use in the home setting. For instance, the environmental factor of electricity blackout leads to electrically powered medical devices failing. Studies show that this type of event causes a dramatic increase in appeal for access to emergency or hospital facilities, and that disaster preparation needs to include the specific needs of patients reliant on electrically driven devices [76–78]. Regarding human factors impacting on safety aspects, one article assessed the suitability of a particular theoretical framework for understanding safety-critical interactions of patients using medical devices in the home [79], while Tennankore et al. [80] described adverse events in home haemodialysis by the use of patients. It was remarkable that none of the articles focused on human factors with regard to the use of medical technologies at home by nurses or other professional caregivers.

#### Design and technological development

Of those articles that focused on this topic, ten reported on the comparison between different types of medical technologies, or their advantages and disadvantages. The comparison of different devices for oxygen therapy was made by two articles [81, 82] and one reported on the comparison of two types of enteral nutrition tubes [83]. Some studies regarding respiratory support considered the process of making a choice between different types of devices [84–86] while one paper considered the conditions for home-based haemo dialysis [87]. A minority, explored the individual characteristics and the clinical applications of several devices for respiratory support [88, 89] and one considered devices for insulin pump therapy [90]. Seven papers discussed the technological development or effectiveness of medical technologies. The testing of devices for external electro-stimulation was described in two papers [91, 92], with the testing of a new design patient lift was subject of one study [93]. Hanada and Kudou [94] explored the current status of electromagnetic interference with medical devices in the home setting, an issue of importance as more devices are considered for home use. The technological development of respiratory support for home use was part of one study [95], as were the possibilities of solar-assisted home haemo dialysis [96]. While the study by Pourtier [97] describes the advantages of analgesia pumps that can be read remotely by nurses, but also emphasizes the central position of a professional nurse in the transfer of information within a multi-disciplinary team.

#### Application with regard to certain diseases or disorders, indications for and extent of use

All articles described several aspects that need to be considered for use, such as clinical characteristics of the patients, indications for the use in the home setting, the technical availability of devices, the extent of their use at home or eventual complications and morbidity. It was important to note that all but one article (*n* = 6) were about children or related to adults with what are usually regarded as paediatric diseases. Results show that the use of AMTs at home among children after hospital discharge is common (in 20%–60% of cases), or is standard for patients with some disorders [98–101]. The timely application of advanced home medical technology benefits patients and can help to reduce respiratory morbidity [102]. Nevertheless, the rate of death of patients with Möbius syndrome using the devices at home was high (30%) [98], as was that of patients with intestinal failure dependent on home parental nutrition therapy in Brazil (75% for 5 years) [103]. The average cumulative survival of children needing home ventilation was found to be between 75 and 90%, depending on the medical diagnosis [104].

#### Policy and management

Three of the papers were concerned with costs and/or reimbursement. The application of medical technologies in the home environment can be cost-effective when compared to institutionalized care [22, 105, 106]. Nevertheless, successful employment of medical technologies in the home necessitates medical guidelines for the indicators for use, careful identification of patients as well as careful planning and attention to details [105–107]. Two studies concerned the dilemma’s for implementation of the technologies in home healthcare and emphasized the importance of cooperation in the chain of key stakeholders to maximize efficiency of high-tech healthcare at home, one with regard to the purchasing policy of medical technologies [108] and one with regard to the interventions of local community service centres and hospitals supporting optimal use of these technologies in the home setting [5].

## Discussion

The use of medical technologies in the home setting has drawn increased attention in health care over the last 15 years, as the feasibility of this type of medical support has rapidly grown. This article systematically reviewed the international literature with regard to the state of the art on this subject, in order to provide a comprehensive overview.

Trend analysis over the period 2000–2015 shows that most research has been conducted about respiratory support, dialysis and oxygen therapy; relatively little about vacuum assisted wound closure and continuous passive motion, and no about decubitus treatment, skeletal traction and UV therapy. A substantial increase in publications was found in the period 2011–2015. Although the number of studies on technologies is indicative of the extent to which they are used in home settings, however, no firm conclusions can be drawn about this.

This review also identified that most research is conducted with regard to ‘user experiences’ of medical technologies in the home, ‘safety, risks, incidents and complications’, and ‘design and technological development of medical technologies’. There have been relatively few studies which have explored the topic of training, instruction and education. Content analysis showed that the use of AMTs in the home setting can have both a positive and a negative psychosocial impact on the patients and their families, and that it has become part of self-management and patient empowerment. Successful use of advanced equipment requires adequate education and training programmes for both patients, informal caregivers and nurses or other professionals. When trying to maximize or assure safety, technological, environmental and human factors have to be taken into account, and it is evident that human factors are the main reason for critical incidents. Studies on the design and technological development of medical technologies emphasize that research is necessary to improve its possibilities and effectiveness. The research found on the application of the technologies focused predominantly on children and the results indicate that the rate of the use of home medical devices among children after hospital discharge is common. Also that when compared to institutionalized care, the application of medical technologies in the home environment can be cost-effective. Much is known, but information on several key issues is limited or lacking.

An important finding was that in almost all the reviewed articles, the study subjects were patients or informal caregivers with very few studies focused on the role and activities of nurses or other professionals as users. This was unexpected as nurses are the main group of users of AMTs at home and they have to transfer knowledge and skills on how to use the devices to patients and other caregivers. Nurses also have a key role in setting up and maintaining collaboration between all actors involved in the process of care with regard to the use of home medical technologies and in giving support to patients and family members in this respect. There is need to initiate further in depth research on AMTs use at home focusing on the role of specifically nurses.

Another interesting result was that, despite the fact that most adverse events with AMTs at home are caused by human factors, hardly any studies conducted on this subject were found. None of the articles focused on related human factors regarding the use by nurses or other professional caregivers, although this is the main user group. Research on this area could contribute to improved patient safety and quality of care. The results also revealed the tension between the advantages and disadvantages of medical technologies as experienced by patients at home. Important aspects needed to promote the benefits include improving the user-friendliness of the devices and attuning their designs for the use in home settings. This emphasizes the importance of professionals (and patient groups) working together with the designers with regard to sharing knowledge and user experiences of the use of AMTs at home in order to improve quality of care and patient safety. This collaboration emerged as of key importance in the successful use of AMTs in the home as well.

Although all included articles were retrieved from academic databases and served our purpose, there was considerable heterogeneity of quality of the studies. Most of the studies have explicitly described their research design, albeit to a greater or lesser extent. On the other hand, there were a few studies that did not even mention their methodological approach, though it could be derived from the description. Most included reviews are of moderate quality. Although findings are almost always described clearly, the search strategy and selection criteria used are often lacking. The quantitative studies are generally well described in different methodological aspects, such as selection of respondents, research design, data collection methods and analyses. Studies of qualitative nature show more variation in the depth with which the design is described. However, almost all qualitative studies have described the research instruments very well, such as semi-structured interviews or questionnaires. Despite the varying quality of the studies, we believe that the whole of different methodological approaches and the relatively large number of included studies (*n* = 87) has yielded a fairly reliable overview on the international state of art concerning various aspects of the use of advanced medical technologies at home. For future research, we recommend to emphasize the development of a more detailed methodological design, zooming in on specific technologies, using large databases or conducting large surveys, and focusing on specific groups of respondents. Both in quantitative and in qualitative studies, a good definition of the research question(s), selection of respondents, development of instruments and analysis of findings, contributes to validity, consistency and neutrality.

Some limitations do have to be taken into account with this review. Although we used the RIVM-definition of ‘advanced medical technology’, not all devices are considered as ‘complex devices’ by nurses in practice. For example, the use of an anti-decubitus mattress in the context of ‘decubitus treatment’ and ‘patient lifting hoists’ are considered by nurses as being of less or lower complexity. However, overall the RIVM-classification was found to be a good starting point, and provided a practical and useful framework from which to work to gain an insight and overview of available medical technologies. Of some of the chosen technologies defined using the RIVM-classification of AMTs, questions do have to be asked as to whether they really are part of the technical skills in nursing process. For example, ‘external electrostimulation’ and ‘continuous passive motion’ are mainly applied by physiotherapists, although with appropriate training nurses can apply them. Then too, devices regarded as only ‘monitoring’ were excluded from the review.

## Conclusions

This systematic review study was designed to fill a gap in the current research by investigating what is known about different aspects of medical technologies used in the home. From the results it is obvious that a wide and growing range of medical technologies are used at home. Different types of technologies have been subject of study, increasingly –also in scope- over the period 2011–2015.

Professional nurses have a central role in the process of homecare which has to be recognized when considering use of AMTs at home. Nurses have to support patients and family caregivers and in consequence have a key role in providing information for, and as a member of multi-disciplinary teams. Closer collaboration by all actors involved in the process of care and feedback of user experiences to the designers is essential for the provision of high quality of care and patient safety.

This review also identified a lack of research exploring the perspectives of nurses in the processes involved in introducing and maintaining technology in homecare. Most of the research has been conducted regarding the experiences of patient experience and how informal caregivers perceive their role in using medical technologies at home. The few studies that were found, demonstrate the need for more research focused on the experiences of nurses working with advanced technologies in the home. The same applies to research on training, instruction and education to use medical technologies, as in these areas too, there was limited available research so here again there is need for further research. Despite the fact that most adverse events with medical technologies in home settings are caused by human factors, our findings also identified a lack of research in this area for nurses.

This study demonstrates that, although there is increasing attention on and recognition of the need for the use of medical technologies in the environment of the home, the research has not kept pace with the advances in care. Subjects such as user experiences of nurses with different technologies, training, instruction and education of nurses and human factors by nurses in risk management and patient safety urgently need to be investigated by further research.

## Abbreviations

AED: Automatic external defibrillator
AMT: Advanced medical technology
CPAP: Continuous positive airway pressure
EC: European Commission
IT: Information technology
NCHS: National Center for Health Statistics
NPV: Negative pressure ventilation
PICCs: Peripherally inserted central catheters
RCT: Randomized Control Trial
RIVM: National Institute for Public Health and the Environment
UV: Ultraviolet
VAD: Ventricular assist device
WHO: World Health Organization
